# Determinants of lungworm specificity in five cetacean species in the western Mediterranean

**DOI:** 10.1186/s13071-021-04629-1

**Published:** 2021-04-12

**Authors:** Rachel Pool, Clara Romero-Rubira, Juan Antonio Raga, Mercedes Fernández, Francisco Javier Aznar

**Affiliations:** Marine Zoology Unit, Cavanilles Institute of Biodiversity and Evolutionary Biology, Science Park, University of Valencia, PO Box 22085, Valencia, 46071 Spain

**Keywords:** Lungworms, Pseudaliidae, Cetaceans, Mediterranean Sea, Host specificity, Parasites

## Abstract

**Background:**

Current data about Pseudaliidae show contrasting patterns of host specificity between congeneric species. We investigated how both contact and compatibility between hosts and parasites contributed to the patterns of lungworm infection observed in a community of five species of cetaceans in the western Mediterranean.

**Methods:**

The lungs of 119 striped dolphins *Stenella coeruleoalba*, 18 bottlenose dolphins *Tursiops truncatus*, 7 Risso’s dolphins *Grampus griseus*, 7 long-finned pilot whales *Globicephala melas*, and 6 common dolphins *Delphinus delphis* were analysed for lungworms. Parasites were identified by morphology and analysis of ITS2 sequences using both maximum likelihood and Bayesian inference methods. Body length was used as a proxy for lungworm species fitness in different hosts and compared with Kruskal-Wallis tests. Infection parameters were compared between cetacean species using Fisher’s exact tests and Kruskal-Wallis tests. Phylogenetic specificity was explored by collating the overall lungworm species prevalence values in hosts from previous surveys in various localities. To explore the relative importance of vertical and horizontal transmission, Spearman’s rank correlation was used to look for an association between host size and lungworm burden. A Mantel test was used to explore the association between lungworm species similarity and prey overlap using dietary data.

**Results:**

*Halocercus delphini* had higher infection levels in striped dolphins and common dolphins; *Stenurus ovatus* had higher infection levels in bottlenose dolphins; and *Stenurus globicephalae* had higher infection levels in long-finned pilot whales. These results are congruent with findings on a global scale. Morphometric comparison showed that the larger nematodes were found in the same host species that had the highest parasite burden. Lungworms were found in neonatal striped dolphins and a Risso’s dolphin, and there was a weak but significant correlation between host size and parasite burden in striped dolphins and bottlenose dolphins. There was also a weak but significant association between prey overlap and lungworm species similarity.

**Conclusions:**

Data indicate that phylogenetic specificity has an important role in governing host–parasite associations, as indicated by the higher infection levels and larger nematode size in certain hosts. However, diet can also influence infection patterns in these preferred hosts and contribute to less severe infections in other hosts. 
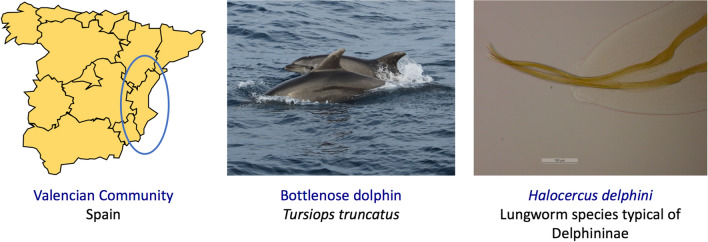

## Background

Adults of all but one of the 29 currently valid species of lungworms of the family Pseudaliidae typically infect the lungs, middle ear and air sinuses of odontocetes worldwide [1, 2]. In heavy infections, lungworms can cause severe health problems such as osseous lesions in the cranial sinuses, blocked airways, verminous pneumonia and secondary bacterial infections, all of which can lead to the stranding or even death of their hosts [1].

Little is known about the life cycles of pseudaliids. Other metastrongyloid nematodes infecting terrestrial or marine mammals have heteroxenous cycles and, depending on the habitat, can use gastropods or oligochaetes as intermediate hosts, or fish as paratenic hosts [1, 3]. In pseudaliids, however, direct evidence on the use of intermediate or paratenic hosts is limited to a single species. Lehnert et al. [4] detected larvae of *Pseudalius inflexus* in dabs *Limanda limanda*, which are common prey of harbour porpoises *Phocoena phocoena* (definitive hosts) in North Sea waters. In other pseudaliids, evidence for trophic transmission is only implied by the absence of infection in unweaned hosts, coupled with the increase in prevalence with host age [1]. However, there is also evidence for vertical transmission from mother to calf at least for three species of *Halocercus* infecting bottlenose dolphins *Tursiops truncatus*, orcas *Orcinus orca*, and striped dolphins *Stenella coeruleoalba* [5–7].

Data about the specificity of pseudaliids are similarly scarce, in part because host sampling is problematic and experimental work virtually impossible. Based on host–parasite records, it would seem that patterns of specificity are contrasting even between congeneric species. For example, *Stenurus minor* seems to be widespread among members of the families Phocoenidae, Delphinapteridae and Delphinidae, whereas *Stenurus arctomarinus* has only been reported in the beluga whale *Delphinapterus leucas* [2, 8]. In addition, the same odontocete species can have strikingly different levels of infection for the same pseudaliid species depending on geographic location. For example, the prevalence of *Halocercus lagenorhynchi* in striped dolphins in Italy, the UK and Costa Rica was 6.67%, 14.3% and 54.2%, respectively [9–11], and this species has never been detected in striped dolphins from the western Mediterranean Sea despite the high sampling effort [7].

Generally speaking, patterns of specificity can be understood as the result of two sequential filters, i.e., contact and compatibility [12–14], which operate at the local community of relevant hosts. Investigating the action of these filters obviously requires data on life cycles (to shed light on patterns of contact) and data on fitness in each host (to shed light on patterns of compatibility). Furthermore, the analysis of specificity must be addressed considering different facets [15], i.e., (1) structural specificity (how parasite populations are distributed among the host species of the local community), (2) phylogenetic specificity (the extent to which these hosts are historically related), and (3) specificity in geographic space (how consistent host use is across a changing host geographic landscape). And, above all, any specificity analysis obviously assumes that parasites are reliably identified.

In this study we investigate, for the first time, patterns of host specificity of the Pseudaliidae in a local community of five cetacean species following the above scheme. First, we combined morphological and molecular methods to verify whether putative nematode species infecting different hosts are indeed conspecific. Second, we looked for evidence of vertical and/or trophic transmission for each lungworm species. Since direct detection of intermediate or paratenic hosts has proven unsuccessful to date (see, e.g., [16]), we investigated the probability of parasite exchange among host species through dietary overlap analysis, assuming that the greater the overlap in the consumption of infected prey species, the higher the probability that the hosts share the same parasites [12–14]. Third, we used proxies to compare fitness of lungworms between host species. Finally, we explored the consistency of host choice in other cetacean communities and estimated the global phylogenetic specificity of the lungworm species based on overall records.

## Methods

### Sample collection

Pseudaliid lungworms were collected from the carcasses of 119 striped dolphins *Stenella coeruleoalba*, 18 bottlenose dolphins *Tursiops truncatus*, 7 Risso’s dolphins *Grampus griseus*, 7 long-finned pilot whales *Globicephala melas*, and 6 short-beaked common dolphins *Delphinus delphis* that were found stranded along the Mediterranean coast of Spain (Valencian Community, between 40°31′00″N, 0°31′00″E and 37°50′00″N, 0°45′42″W) between 1982 and 2019. In the case of the striped dolphins, we excluded individuals from 1990 and 2007 because they had suffered a *Morbillivirus* outbreak, which could produce a confounding effect on parasite abundance. Only carcasses in preserved states 1–3 [17] were selected for analysis.

During the necropsy, the animals were measured and sexed; their cranium was disarticulated from the spine and cranial sinuses were examined for parasites, and the lungs were removed and stored at −20 °C. Only one lung per individual, randomly selected, was used in this study; the other was saved for a future study on microhabitat selection. After thawing, the airways of each lung were cut from the trachea to the caudal apex and rinsed over a 0.2 mm sieve.

All fragments and whole worms were collected, cleaned in 0.9% saline, and fixed and preserved in 70% or 90% ethanol for morphological and molecular analysis, respectively. Specimens were examined under a light microscope and identified following Delyamure [18]. Due to the tendency of some lungworm species to embed their anterior ends in the parenchyma, it was difficult to extract worms whole. Thus, the total worm number per lung was estimated as the number of complete worms, or incomplete worms with their caudal end intact. In the case of pilot whales, only presence/absence data for lungworm species could be recorded.

### Molecular identification of species

Extraction of genomic DNA using the DNeasy^®^ Blood and Tissue Kit (QIAGEN, Hilden, Germany) was successful for 21 out of 26 selected lungworms from 12 hosts (Table 1). The second internal transcribed spacer (ITS2) of nuclear ribosomal DNA was chosen as the barcoding genetic marker due to its reliability in nematode species identification [4, 19]. The primers PseuITS2F (5’-CCT TCG GCA CAT CTT GTT CA-3’) and PseuITS2R (5’-GGG TAA TCA CAT CTG AGT TCA-3’) [19] were used at a concentration of 5 pmol/μl. The polymerase chain reaction (PCR) reaction mixtures had a final volume of 20 μl, with 2 μl DNA, 4.8 μl PCR water, 1.6 μl of each primer and 10 μl MyFiTM DNA Polymerase (Bioline, Meridian Life Science, Inc., Taunton, MA, USA). After an initial heat-activation step of 94 °C for 3 min, the reaction consisted of 39 cycles of 94 °C for 1 min, 58 °C for 1 min and 72 °C for 1 min, followed by a final step at 72 °C for 5 min. Positive and negative (no DNA) controls were used in each PCR.

**Table 1:** GenBank accession numbers of sequences of second internal transcribed spacer (ITS2) used in the phylogenetic analyses of 20 individuals of three species of the family Pseudaliidae Railliet and Henry 1909

GenBank Acc. no.	Sequence ID	Seq. length (bp)	Family	Host^a^	Locality
DQ408626	*Heligmosomum mixtum*		Heligmosomidae	Cg	Poland
DQ679968	*Hovorkonema variegatum*		Syngamidae	Ggu	Germany
AY491979	*Otostrongylus circumlitus*		Crenosomatidae	Pv	NE Atlantic
FJ787304	*Parafilaroides gymnurus*		Filaroididae	Pv	NE Atlantic
FJ787301	*Halocercus invaginatus*		Pseudaliidae	Pp	NE Atlantic
FJ767935	*Pseudalius inflexus*		Pseudaliidae	Pp	NE Atlantic
AY464532	*Torynurus convolutus*		Pseudaliidae	Pp	NE Atlantic
FJ787302	*Stenurus minor*		Pseudaliidae	Pp	NE Atlantic
FJ787303	*Stenurus globicephalae*		Pseudaliidae	Gm	NE Atlantic
MW192227	*Stenurus globicephalae* Gm1-1	496	Pseudaliidae	Gm	Mediterranean
MW192228	*Stenurus globicephalae* Gm1-2	507	Pseudaliidae	Gm	Mediterranean
MW192226	*Stenurus globicephalae* Gg	530	Pseudaliidae	Gg	Mediterranean
MW192229	*Stenurus ovatus* Sc	530	Pseudaliidae	Sc	Mediterranean
MW192230	*Stenurus ovatus* Tt1-1	517	Pseudaliidae	Tt	Mediterranean
MW192231	*Stenurus ovatus* Tt1-2	513	Pseudaliidae	Tt	Mediterranean
MW192225	*Halocercus delphini* Gg	513	Pseudaliidae	Gg	Mediterranean
MW192224	*Halocercus delphini* Tt	525	Pseudaliidae	Tt	Mediterranean
MN747504	*Halocercus delphini* Dd1-1	523	Pseudaliidae	Dd	NE Atlantic
MN747503	*Halocercus delphini* Dd2-1	487	Pseudaliidae	Dd	Mediterranean
	*Halocercus delphini* Sc1-1	479	Pseudaliidae	Sc	Mediterranean
	*Halocercus delphini* Sc2-1	472	Pseudaliidae	Sc	Mediterranean
	*Halocercus delphini* Sc3-1	437	Pseudaliidae	Sc	Mediterranean
MN747502	*Halocercus delphini* Sc3-2	555	Pseudaliidae	Sc	Mediterranean
	*Halocercus delphini* Sc3-3	521	Pseudaliidae	Sc	Mediterranean
	*Halocercus delphini* Sc3-4	385	Pseudaliidae	Sc	Mediterranean
	*Halocercus delphini* Sc4-1	413	Pseudaliidae	Sc	Mediterranean
	*Halocercus delphini* Sc4-2	540	Pseudaliidae	Sc	Mediterranean
	*Halocercus delphini* Sc4-3	449	Pseudaliidae	Sc	Mediterranean
	*Halocercus delphini* Sc4-4	536	Pseudaliidae	Sc	Mediterranean

*Heligmosomum mixtum* Schulz, 1929, *Hovorkonema variegatum* Creplin, 1849, *Otostrongylus circumlitus* Railliet, 1899, and *Parafilaroides gymnurus* Railliet, 1899, were used as outgroups. In samples of *Stenurus globicephalae*, *Stenurus ovatus*, and *Halocercus delphini*, ID numbers stand for individual host and individual specimen, respectively

^*a*^Host codes: Cg: *Clethrionomys glareolus* Schreber; Ggu: *Grus grus* Linnaeus; Pv: *Phoca vitulina* Linnaeus; Pp: *Phocoena phocoena* Linnaeus; Gm: *Globicephala melas* Traill; Gg: *Grampus griseus* Cuvier; Sc: *Stenella coeruleoalba* Meyen; Dd: *Delphinus delphis* Linnaeus

Aliquots of 2 μl of each amplicon were mixed with 2 μl of loading dye and run on agarose gel (1% gel of 0.4 g agar powder and 40 ml TE buffer) stained with GelRed^®^ Nucleic Acid Gel Stain (Biotium, Hayward, CA, USA) for electrophoresis. The bands were visualized and photographed using an ultraviolet light hood. After purification with the Nucleospin^®^ PCR and Gel Purification Clean-up kit (Machery-Nagel, Düren, Germany), amplicons were sent to Macrogen Europe (Amsterdam, Netherlands) for sequencing with the same primers used for the PCR reactions.

Nucleotide sequences from both strands were used to assemble consensus sequences with Geneious R7 (https://www.geneious.com). The sequences of *H. delphini* from *D. delphis* and *S. coeruleoalba* were previously published in Pool et al. [20].

Sequences were aligned using MAFFT [21] with published GenBank sequences for the Pseudaliidae, with *Heligmosomum mixtum* Schulz, 1929, and *Hovorkonema variegatum* Creplin, 1849, being designated as outgroups (see [20], Table 1). The resulting alignment was edited to cut significant gaps using the heuristic method in trimAl v1.2 [22] and then analysed using maximum likelihood (ML) and Bayesian inference (BI) methods. Both methods used the Hasegawa-Kishino-Yano nucleotide substitution model with a gamma rate of inversion, which was selected according to the Akaike information criterion (AIC) by jModelTest [23, 24]. The ML analysis was conducted in MEGA X [25] with 500 bootstrap replications. The BI analysis was performed with MrBayes 3.2.5 [26], with posterior probability values calculated by four simultaneously running Markov chains using 1,200,000 generations. Trees were sampled every 1000th generation, and an average standard deviation of split frequencies < 0.01 was used as an indication that convergence had been achieved. A total of 25% of the trees were discarded as burn-in.

Tree topologies of the ML and BI analyses were checked for congruence using the FigTree v.1.4.4 program [27]. Pairwise distances were also calculated with MEGA X [24], with complete deletion of gaps.

### Specificity analysis

We set the 95% confidence interval for prevalence of lungworms using Sterne’s exact method and, for mean intensity and mean abundance, the bias-corrected and accelerated bootstrap method with 20,000 replications [28]. Prevalence and intensity of lungworm species were compared between host species with Fisher’s exact and Kruskal-Wallis tests, respectively. In pairwise comparisons, *P* values were corrected by the Holm-Bonferroni sequential method with *α* = 0.05. Note, however, that the power of some tests was low due to small host sample sizes.

To investigate the life cycles of lungworms, dolphins of each species were separated into three age categories, i.e. neonate, suckling and weaned individual, based on published data (Table 2). Then, for each lungworm species, the possibility of vertical and/or horizontal transmission was explored by recording infection in neonates (suggestive of vertical transmission) and/or weaned individuals (suggestive of horizontal transmission), respectively. When sample size allowed, we also used one-tailed Spearman correlation to test for an increase in worm abundance with host body length (excluding neonates), since larger hosts could have more infection opportunities due to higher food consumption [1].

**Table 2:** Age groups of each cetacean host species based on body lengths from previously published literature

Host species	Body length (cm)			Locality	References
	Neonate	Suckling	Weaned		
*Stenella coeruleoalba*	< 93	93–165	≥ 165	Mediterranean Sea	Pool et al. [7]
Striped dolphin					
*Tursiops truncatus*	< 115	115–170	≥ 170	NW Atlantic Ocean	Read et al. [29]
Bottlenose dolphin					Struntz et al. [30]
*Grampus griseus*	< 140	140–230	≥ 230	Mediterranean Sea	Raduan et al. [31]
Risso's dolphin				NW Atlantic Ocean	Bloch et al. [32]
*Delphinus delphis*	< 93	93–160	≥ 160	Atlantic Ocean	Westgate and Read [33]
Common dolphin					

The influence of trophic transmission on patterns of infection was further explored by examining whether there was a significant association between diet and lungworm fauna among host species. Dietary data were available from the very same samples of dolphins used for the present parasitological analysis [34], [35], [36], unpublished data], except long-finned pilot whales, for which we chose data from the nearest geographical region, the Iberian Atlantic [37]. We did not expect fundamental differences in diet between the Atlantic and Mediterranean populations of pilot whales because prey data were dealt with at the genus level. For each dolphin species, we selected all prey taxa that had been identified at least up to genus level (in all dolphin species, they represented ≥ 90% of total individual prey), and obtained the matrix of pairwise percent similarity index (PS) of diet between dolphin species:

$${\text{PS}}_{ij} = \, \Sigma_{k} {\text{min }}\left( {y_{ki} , \, y_{kj} } \right)/\left( {\Sigma_{k} y_{ki \, + } \Sigma_{k} y_{kj} } \right),$$

where min (*y*_*ki*_, *y*_*kj*_) is the minimum value of frequency of occurrence (%) in each pair of shared prey between dolphin species *i* and *j* summed across *k* shared species, and (Σ_k_ *y*_*ki* +_ Σ_*k*_ *y*_*kj*_) is the sum of the frequency of occurrences of all prey in both dolphin species. Likewise, we obtained the PS matrix for lungworm fauna based on prevalence data. The potential association between the prey and lungworm matrices was tested with the Mantel test [38].

We used adult female worm size to examine the potential effects of each dolphin species on the fitness of each lungworm species [39] (i.e., the compatibility filter). We collected as many intact adult female worms as possible up to 10 individuals per lungworm and dolphin species. Unfortunately, sample sizes were smaller in some cases because not enough (suitable) specimens were found. The selected females were put on a Petri dish with saline and covered with a microscope slide; their length was measured from drawings obtained with the aid of a drawing tube connected to a stereomicroscope (×100). Depending on the lungworm species and number of available specimens, worm length was compared with one-way analysis of variance (ANOVA) with post hoc Šidák test, *t* test for independent samples or one-sample *t* tests (see the “Results” Section).

To explore phylogenetic specificity, we searched available parasitological surveys in the bibliography that recorded any of the lungworms specie found in the present study. Then, we obtained overall values of prevalence per host species (i.e., total number of infected hosts / total host sample size, regardless of locality). To control for sampling effort, these prevalence values were weighed by relative sample size of each host species. Weighed values were placed in the most recent phylogenetic tree of the order Cetacea [40] and interpreted visually. On the other hand, the geographical consistency in host use was examined based on all parasitological surveys of local cetacean communities in which (1) at least one of the lungworm species detected in this study was reported, and (2) at least three host species of different genera were analysed for lungworms.

Quantitative Parasitology version 3.0 software was used to calculate parasite burden parameters [41]. The Mantel test was carried out with the free software PASSaGE version 2 [38]. All other statistical analyses were performed with R [42]. The significance level was set at *P* < 0.05.

## Results

A total of 3 of 7 long-finned pilot whales, 4 of 7 Risso’s dolphins, 9 of 18 bottlenose dolphins, all 6 common dolphins, and 73 of 119 striped dolphins were infected with lungworms. Three species of lungworm were morphologically identified in the whole sample of hosts, namely, *Halocercus delphini, Stenurus ovatus* and *S. globicephalae* (Table 3).

**Table 3:** Infection parameters (with 95% CI in parentheses, and range in brackets) of species of the Pseudaliidae found in five cetacean species stranded on the Spanish Mediterranean coast between 1987 and 2019

Host species	*n*	*Halocercus delphini*			*Stenurus ovatus*			*Stenurus globicephalae*		
		*P*	MA	MI	*P*	MA	MI	*P*	MA	MI
*Stenella coeruleoalba*	119	61.3 (52.1–69.8)	25.70 (18.30–37.30)	41.8 (30.1-59.0) [1–278]	5.9 (2.8–11.7)	0.084 (0.03–0.17)	1.4 (1.0–2.0) [1–3]	0.0 (0.0–3.2)	0.00	0.0
*Tursiops truncatus*	18	27.8 (11.6–52.9)	1.11 (0.28–3.00)	4.0 (1.8–7.3) [1–10]	33.3 (15.6–58.6)	5.61 (1.56–14.80)	16.8 (6.1–32.2) [2–48]	0.0 (0.0–18.5)	0.00	0.0
*Delphinus delphis*	6	100.0 (58.9–100.0)	20.80 (8.33–50.80)	20.8 (8.5–52.6) [4–71]	16.7 (0.9–58.9)	0.167 (0.00–0.33)	1.0	0.0 (0.0–41.1)	0.00	0.0
*Grampus griseus*	7	42.9 (12.9–77.5)	1.43 (0.14–4.71)	3.3 (1.0–5.6) [1–8]	0.0 (0.0–37.7)	0.00	0.0	57.1 (22.5–87.1)	0.71 (0.14–1.14)	1.2 (1.0–1.5) [1, 2]
*Globicephala melas*	7	0.0 (0.0–37.7)	0.00	0.0	0.000 (0.000–0.377)	0.00	0.0	42.9 (12.9–77.5)		

*P*, prevalence (%); MA, mean abundance; MI, mean intensity; *n*, number of hosts analysed. Blank cells indicate values for which data were unavailable

### Molecular analysis

Contigs of the ITS2 region were able to be obtained from 21 lungworms. After trimming, the alignment was 303 bp long; 8 sequences were uploaded to GenBank (Table 1). We obtained three partial ITS2 sequences of *Stenurus ovatus* (513–550 bp long), one from a striped dolphin and two from a bottlenose dolphin. Comparison of pairwise divergence in the alignment showed that all three sequences were identical.

Four partial ITS2 sequences of *Stenurus globicephalae* were obtained, one from Risso’s dolphin and three from the long-finned pilot whale. One of the long-finned pilot whale sequences was discarded from the alignment because it was too short (248 bp long). The sequence from Risso’s dolphin was identical to one of the sequences from the pilot whale; the second pilot whale sequence had a pairwise divergence of 0.012.

Fourteen partial ITS2 sequences of *Halocercus delphini* were obtained. Uncorrected pairwise distances in the alignment varied between 0.000 and 0.060 (Additional file 1: Table S1).

**Fig. 1:**
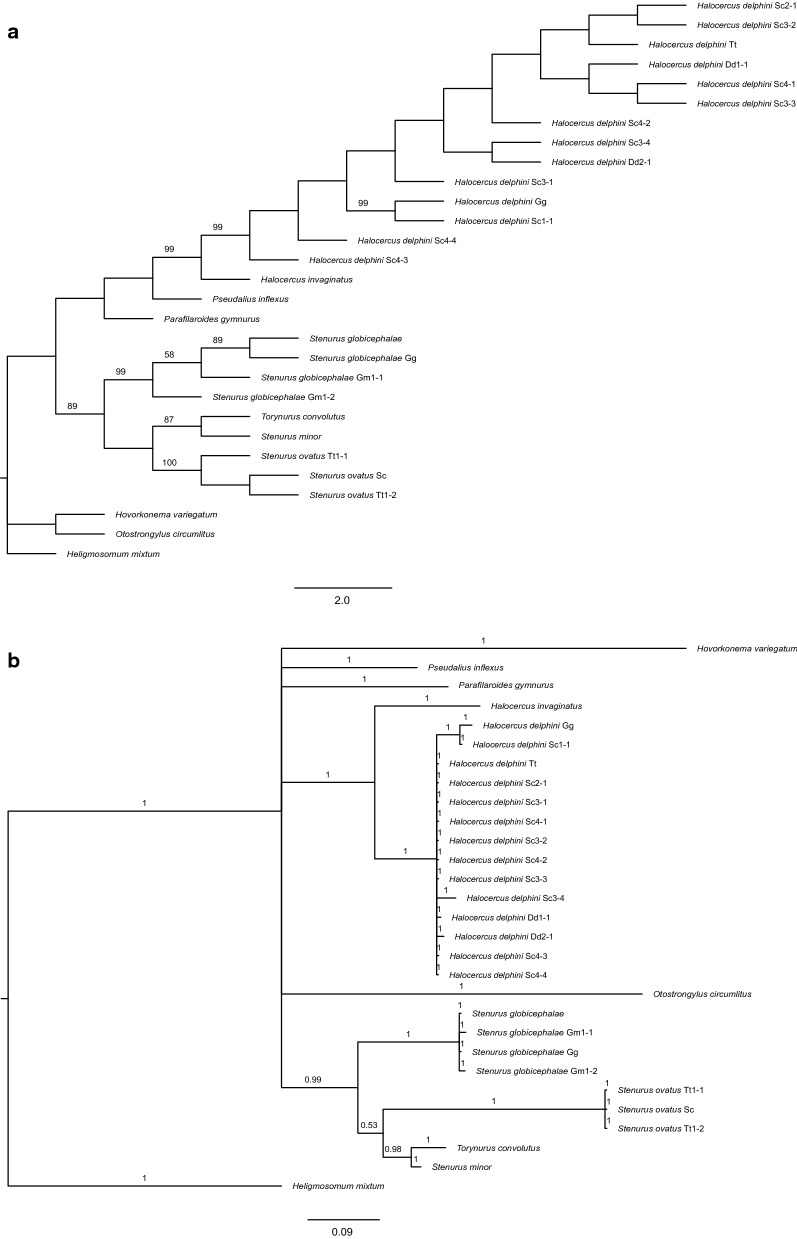
**a** Maximum likelihood analysis of the phylogenetic relationships of various pseudaliid lungworm samples from cetaceans in the western Mediterranean in relation to other members of the Metastrongyloidea superfamily using second internal transcribed spacer sequences. *Heligmosomum mixtum* from the family Heligmosomatidae and *Hovorkonema variegatum* from the family Syngamidae were used as the outgroups. Nodal support is indicated by bootstrap values; bootstrap values less than 50% are not shown. The scale bar indicates the number of nucleotide substitutions per site. **b** Bayesian inference analysis (BI) of the phylogenetic relationships of various pseudaliid lungworm samples from cetaceans in the western Mediterranean in relation to other members of the Metastrongyloidea superfamily using second internal transcribed spacer sequences. *Heligmosomum mixtum* from the family Heligmosomatidae and *Hovorkonema variegatum* from the family Syngamidae were used as the outgroups. Nodal support is indicated by BI posterior probabilities; posterior probabilities less than 0.7 are not shown. The scale bar indicates the number of nucleotide substitutions per site

The ML and BI analyses produced trees with dissimilar topology regarding the outgroups, but in both trees all samples of *Stenurus ovatus* appeared as a highly supported clade (ML: 100%; BI: 1), with *Stenurus minor* and *Torynurus convolutus* as the sister taxa (Fig. 1). All samples of *Stenurus globicephalae*, including the previously published sequence from GenBank, also appeared together in a highly supported clade (ML: 99%; BI: 1) in both trees (Fig. 1). *Halocercus invaginatus* appeared as the sister taxon of a highly supported clade in which all of the samples of *Halocercus delphini* (regardless of host species) clustered together (ML: 99%; BI: 1) (Fig. 1).

### Specificity analysis

*Halocercus delphini* was found in the lungs of all host species except pilot whales (Table 3). The prevalence (Fisher’s exact test, *P* = 0.004) differed significantly between host species, being significantly higher in striped and common dolphins compared with bottlenose dolphins and pilot whales. The intensity also differed among infected species (Kruskal-Wallis test, χ^2^ = 10.37, 3 *df*, *P* = 0.016), with significantly higher values in striped dolphins compared with those from bottlenose and Risso’s dolphins (Tables 3, 4).

**Table 4:** Nominal (P) and sequential Bonferroni-corrected (B-P) probability values of pairwise *post hoc* comparisons of Fisher’s test (for prevalence) and Kruskal-Wallis (K-W) test (for intensity) of lungworm species infecting five cetacean species from the western Mediterranean

Pair	*Halocercus delphini*				*Stenurus ovatus*				*Stenurus globicephalae*	
	Fisher		K-W		Fisher		K-W		Fisher	
	P	B-P	P	B-P	P	B-P	P	B-P	P	B-P
Sc–Tt	0.010*	0.070	0.037*	0.185	0.002*	0.022*	0.005*	0.015*	1	1
Sc–Dd	0.084	0.422	0.754	1	0.376	1	0.739	0.739	1	1
Sc–Gg	0.434	0.868	0.011*	0.055	1	1			< 0.0001*	< 0.0001*
Sc–Gm	0.002*	0.016*			1	1			< 0.0001*	< 0.0001*
Tt–Dd	0.003*	0.027*	0.121	0.363	0.626	1	0.077	0.154	1	1
Tt–Gg	0.640	0.640	0.92	0.92	0.137	1			0.003*	0.022*
Tt–Gm	0.274	0.823			0.137	1			0.003	0.025
Dd–Gg	0.070	0.419	0.069	0.276	1	1			0.070	0.350
Dd–Gm	0.001*	0.006*			1	1			0.070	0.350
Gg–Gm	0.193	0.772			1	1			1	1

Note that intensity data were not available for one of the two species (Gm) infected with *Stenurus globicephalae*; thus no intensity comparison was made in this case. Abbreviations: Dd *Delphinus delphis*; Gg *Grampus griseus*; Gm *Globicephala melas*; Sc *Stenella coeruleoalba*; Tt *Tursiops truncatus*. *Denotes significance level of *P* < 0.05

*Stenurus ovatus* was found in the lungs of bottlenose, common and striped dolphins, and both the prevalence (*P* = 0.004) and the intensity (χ^2^ = 8.89, 2 *df*, *P* = 0.012) were significantly higher in bottlenose dolphins than striped dolphins (Table 4); a single worm was found in one common dolphin (Table 3, 4). Finally, *S. globicephalae* was detected in the lungs of Risso’s dolphins and the lungs and cranial sinuses of long-finned pilot whales (Table 2). The statistical analysis revealed significant differences in prevalence between these hosts and bottlenose and striped dolphins (Table 4).

Five neonates of striped dolphin harboured *H. delphini* (range of intensity: 1–80), and a single neonate of Risso’s dolphin (136 cm long) harboured 8 individuals of *H. delphini* and 1 of *S. globicephalae* (Table 5). Lungworms of all species also occurred in both suckling and weaned hosts. The abundance of *H. delphini* was positively, but weakly, associated with host body length in the striped dolphin (*r*_s_ = 0.152, *n* = 114, one-tailed *P* = 0.053); the association was much stronger in the case of *S. ovatus* in bottlenose dolphins (*r*_s_ = 0.677, *n* = 18, *P* = 0.001).

**Table 5:** Prevalence (95% CI) of lungworms per age group in five cetacean species from the western Mediterranean

Parasite	Host	Age group					
		Neonate		Unweaned		Weaned	
		*n*	Prevalence	*n*	Prevalence	*n*	Prevalence
*Halocercus delphini*	Sc	5	20 (10.0–65.7)	30	63.3 (44.9–78.7)	84	63.1 (52.4–72.8)
	Tt	–	–	2	100	16	18.8 (5.2–43.6)
	Dd	–	–	1	100	5	100 (50.0–100)
	Gg	1	100	–	–	6	33.3 (6.3–72.9)
*Stenurus ovatus*	Sc	–	–	30	3.3 (0.2–17.7)	84	7.1 (3.2–14.7)
	Tt	–	–	2	0	16	37.5 (17.8–62.8)
	Dd	–	–	1	0	5	20.0 (1.0–65.7)
*Stenurus globicephalae*	Gg	1	100	–	–	6	50.0 (15.3–84.7)
	Gm	1	0	1	100	5	40.0 (7.7–81.1)

*CI* Confidence Interval

There was non-negligible overlap in the diet of the five cetaceans under study; shared prey included both fish and cephalopods in all cases (Additional file 2: Table S2). The overlap was particularly high between the two predominantly teuthophagous species (pilot whales and Risso’s dolphin), the two predominantly piscivorous species (bottlenose dolphin and common dolphin), and the species with a mixed diet (the striped dolphin) with respect to the remaining ones (Table 6). A weak but significant positive association between diet overlap and similarity was detected in the lungworm fauna (Table 6, Mantel test, *r*_M_ = 0.57, one-tailed *P* = 0.011).

**Table 6:** Matrices of average percent shared prey taxa (at least at genus level), percent similarity of diet (PSD) and percent similarity of lungworm fauna based on prevalence (PSL) among five odontocete species from the western Mediterranean (see the text for details)

	Gm	Gg	Tt	Dd
Percent shared taxa				
Gm				
Gg	38.5			
Tt	36.5	29.7		
Dd	35.1	22.3	47.1	
Sc	33.9	47.7	41.2	48.2
PSD				
Gg	20.1			
Tt	12.4	11.8		
Dd	13.3	10.2	18.2	
Sc	14.6	23.6	15.0	21.1
PSL				
Gg	27.6			
Tt	0	16.9		
Dd	0	23.5	18.2	
Sc	0	28.7	27.0	35.4

Data on body length of female lungworms were difficult to obtain from some hosts due to very low infection levels and/or scarcity of intact worms. A total of 10 individuals of *H. delphini* could be measured from striped dolphins (mean ± SD [mm]: 58.9 ± 14.3), 5 from common dolphins (48.0 ± 11.1), 2 from bottlenose dolphins (20.1 ± 3.7), and none from Risso’s dolphin. Mean length values differed significantly (one-way ANOVA, *F*_(2,11)_ = 7.48, *P* = 0.009); the probability associated with pairwise comparisons (Šidák test) was *P* = 0.008 (striped vs bottlenose dolphins); 0.064 (common vs bottlenose dolphin) and *P* = 0.378 (striped vs common dolphin). In the case of *S. ovatus*, 10 females could be measured from bottlenose dolphins (22.9 ± 4.5), 2 from striped dolphin (9.2 ± 11.0) and none from common dolphin. Mean length values differed significantly between bottlenose and striped dolphin (*t* test, *t* = 3.20, 10 *df*, *P* = 0.009). Finally, the number of measured females of *S. globicephalae *were 10 from pilot whales (46.1 ± 4.7) and just 1 from Risso’s dolphin (11.4). The latter value was significantly smaller than the length range observed for pilot whales (one-sample *t* test, *t* = 23.5, 9 *df*, *P* < 0.0001).

**Fig. 2:**
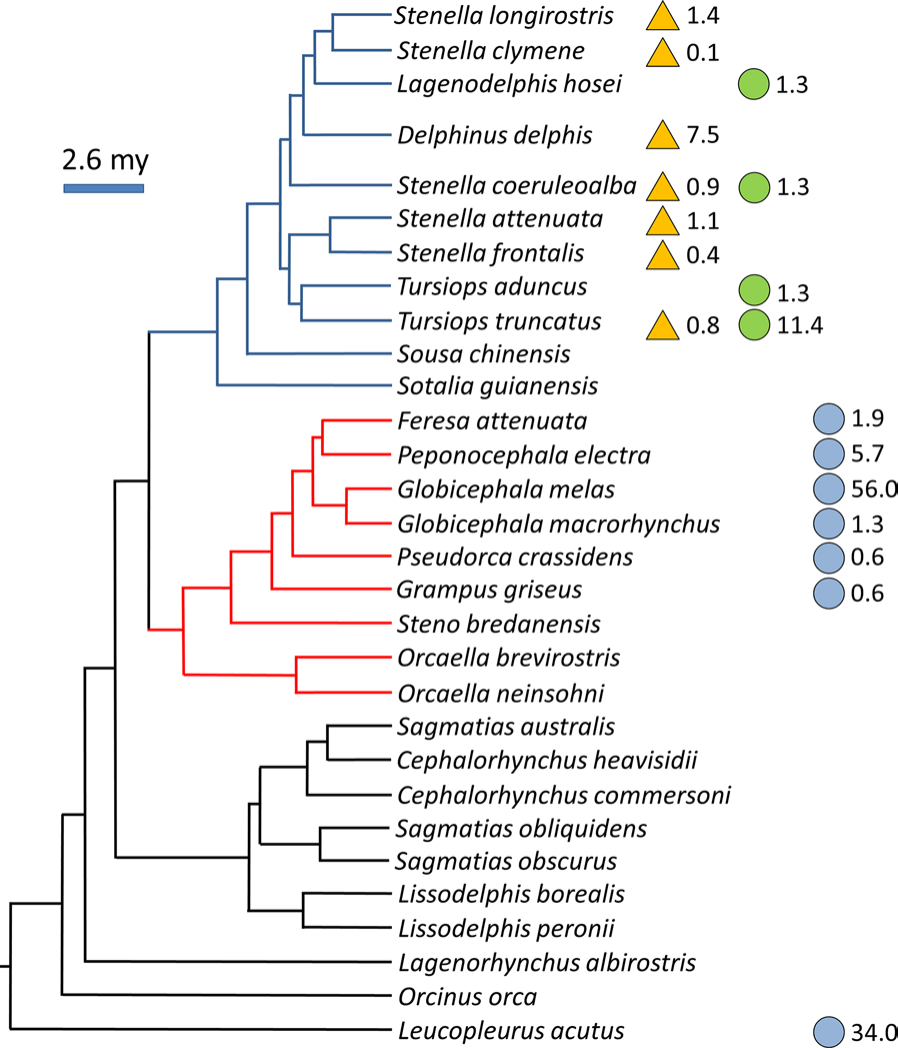
Weighed prevalence values obtained from surveys conducted in different localities [10, 61–65] of the three lungworm species found in this study placed on the phylogenetic tree of the order Cetacea [40]. Abbreviation: my, millions of years ago. Key:  , *Halocercus delphini*;  , *Stenurus ovatus*;  , *Stenurus globicephalae*

Data from previous field surveys reporting quantitative data on any of the lungworm species detected in this study are shown in Additional file 3: Table S3; further qualitative records did not increase the number of infected species (data not shown). All records concern the Atlantic [43–49] and Pacific Oceans [50–58] and the Mediterranean Sea [59, 60]. *Halocercus delphini* and *S. globicephalae* were found in 7 species and *S. ovatus* in 4 (Fig. 2). When placed in a phylogenetic context, both *H. delphini* and *S. ovatus* were observed to be restricted to the clade of the Delphininae, and *S. globicephalae* to the clade of the Globicephalinae, with the notable exception of the infection of *Leucopleurus (Lagenorhynchus) acutus*, which is considered a basal taxon in the Delphinidae (Fig. 2). Data on weighed prevalence indicated that, based on available evidence and sampling effort, *H. delphini* is clearly associated with common dolphin, *S. ovatus* with bottlenose dolphin, and *S. globicephalae* with long-finned pilot whales and *L. acutus* (Fig. 2).

We collected information from six field surveys encompassing ≥ 3 spp. of delphinids, of which at least one harboured some of the lungworm species detected in this study (Additional file 4: Table S4). Interestingly, in sympatric populations of common dolphins, bottlenose dolphins and *Stenella* spp., *H. delphini* did not exhibit a consistent pattern of infection. For instance, in the North Atlantic, this lungworm occurred in common dolphins but not in striped dolphins, whereas in the eastern Pacific it was found in *Stenella* spp. but not in common dolphins (Additional file 3: Table S3). Although host sample sizes were smaller, *S. globicephalae* and *S. ovatus* appeared to be associated with *Globicephala* spp. and the bottlenose dolphin, respectively (Additional file 3: Table S3).

## Discussion

Using their morphological characteristics, we identified three species of lungworms from the five species of cetaceans examined in this study: *Halocercus delphini*, *Stenurus globicephalae* and *S. ovatus*. The host–parasite associations observed reflect the results of previous parasitological surveys in the area, but this study also provides a new host record for *H. delphini* in Risso’s dolphin and a new host record of *S. ovatus* in the short-beaked common dolphin. Only one other lungworm species has been detected in delphinids in the Mediterranean, *Stenurus minor*, which was apparently found in the stomachs of striped dolphins from the Eastern Mediterranean, perhaps as the result of accidental swallowing [66]. These patterns strongly suggest that we likely detected all the lungworm species infecting delphinids in the western Mediterranean.

Although there was an apparent effect of host species on the morphometrics of the worms (see worm size below), the results of the molecular analysis of the ITS2 barcoding region of ribosomal DNA was congruent with species identification based on morphological descriptions. All specimens of each morphospecies clustered together, with high support, and sequences were either identical or showed high levels of similarity, regardless of the host species in which worms were found. Most importantly, the intraspecific variations in pairwise distance were similar to those obtained for other metastrongyloids [67] and did not follow a pattern determined by host species and/or host specimen. Admittedly, the placement of the outgroups varied between ML and BI trees, and in both analyses, *Parafilaroides gymnurus* was placed firmly within the Pseudaliidae and *Torynurus convolutus* was the closest relative to *Stenurus minor* and placed in the *Stenurus* clade. These phylogenetic incongruences in the Pseudaliidae have previously been noted [4, 68], but we think that these taxonomic issues would have a negligible effect on species-level differences.

Each parasite was found in multiple hosts, and yet despite small sample sizes and reduced power of statistical tests, each lungworm species showed a tendency to concentrate in one or more host species in particular. *Halocercus delphini* was found in all hosts except the long-finned pilot whale, but the abundance and prevalence of this parasite were significantly higher in striped dolphins and common dolphins, demonstrating a clear host preference. By contrast, species of *Stenurus* seemed more host-specific in this study. In the case of *S. ovatus*, which was found in only three of the four cetacean species examined, the main definitive host appears to be the bottlenose dolphin, which, despite its small sample size, had a significantly higher prevalence and abundance of *S. ovatus* than the striped dolphin. *Stenurus globicephalae* appeared to be the most specific of the lungworms found in this study, having been detected in only the Risso’s dolphin and the long-finned pilot whale, with a significantly higher prevalence in the latter.

These results are congruent with the findings of the comparative morphometric analysis of female nematodes, despite the admittedly small sample sizes for some comparisons meaning that the statistical tests lacked substantial power. Females of *Halocercus delphini* were significantly longer in the striped dolphin and the common dolphin, whereas those of *S. ovatus* were longer in the bottlenose dolphin, and females of *S. globicephalae* were longer in the long-finned pilot whale. Nematode body length is correlated with fecundity [39], and therefore a conservative interpretation would be that there is a correlation between nematode fitness (and therefore host–parasite compatibility) and infection burden.

When the observed parasitic burden patterns are analysed within a wider phylogenetic and geographic context using previous data, we can readily interpret them assuming the operation of phylogenetic specificity. *Halocercus delphini* was associated with host species from the clade *Delphinus* + *Stenella* + *Tursiops*. Interestingly, the highest overall prevalence value was found in common dolphin, such as we observed in our area. However, the importance of species of *Delphinus* and *Stenella* in supporting populations of this parasite seems to vary among localities, with bottlenose dolphin playing a secondary role, if at all (Additional file 4: Table S4). In contrast, bottlenose dolphins appeared to be most associated with *S. ovatus* within the same *Delphinus* + *Stenella* + *Tursiops* clade. On the other hand, *S. globicephalae* was geographically widespread only in species of the Globicephalinae and the Atlantic white-sided dolphin, *Leucopleurus acutus*, being most frequently found in long-finned pilot whales.

The consistency of these findings with ours suggests that the infection patterns observed in this study are regulated by host–parasite compatibility, as the parasites may have limited ability to establish themselves in unsuitable hosts. However, the infections of lungworm species outside of their preferred subset of hosts (as observed in this study) are likely driven by contact through shared diet, as supported by the weak but significant positive association between host diet overlap and similarity in the lungworm fauna. Additionally, the significant positive correlations of lungworm abundance with host size in the case of *H. delphini* in striped dolphins and *S. ovatus* in bottlenose dolphins show that trophic transmission can also influence lungworm infection patterns within their preferred hosts. It is unlikely that these correlations are the result of long-lasting infections acquired as neonates, as there were no infected neonates of bottlenose dolphins, but also because the prevalence of infection in both species of lungworms was higher in weaned individuals, many of which were adults. Older (larger) dolphins would have had more time to have been exposed to infected prey and would be more likely to consume infected prey due to higher metabolic requirements [7].

An interesting question is the extent to which vertical transmission could help reinforce the observed patterns of specificity. Currently, proof of vertical transmission exists for species of *Halocercus* [5–7] and *Stenurus arctomarinus* [8]. Evidence of *H. delphini* being capable of vertical transmission in striped dolphins from the Mediterranean has been published previously from part of this same population [7], and this study also provides new evidence to that effect, with the finding of *H. delphini* in a Risso’s dolphin neonate (*n* = 8). *Stenurus globicephalae* (*n* = 1) was also found in the same individual. This is the first record of vertical transmission of *H. delphini* in a Risso’s dolphin, and also the first record of *S. globicephalae* being vertically transmitted. The means of this mode of transmission are still unclear, as there are three proposed non-exclusive mechanisms: inhalation of infected spray (i.e., aerosol), consumption of infected milk (i.e., lactogenic transmission) or transplacental infection [5, 69, 70].

The fact that the neonatal Risso’s dolphin harboured two species of lungworm could mean that both were transmitted the same way, or equally, it could mean that each species was transmitted via a different route. Unfortunately, there is no definitive proof for any of the proposed mechanisms, or for their relative role in vertical transmission. Considering the absence of lungworms in neonates in comparison to the frequency of infection of weaned individuals of the other three cetaceans, it is likely that horizontal transmission plays a more important role than vertical transmission in the initial infections of the hosts. However, it is difficult to assess the relative importance of vertical versus horizontal transmission in the life cycle of pseudaliids due to the lack of quantitative data in neonates, which are typically under-represented in samples of dolphins used for parasitological analysis.

## Conclusion

In conclusion, this is the first study in which a quantitative assessment of host–lungworm specificity has been attempted in a local community of cetaceans. Two new host records are reported for *H. delphini* and *S. ovatus*. Available data suggest that phylogenetic specificity is a key driver of host–parasite associations: *Halocercus delphini* exhibits a preference for striped dolphins and common dolphins, and these are its preferred hosts in a global context, whereas *Stenurus ovatus* and *S. globicephalae* similarly demonstrate obvious host preference for the bottlenose dolphin and the long-finned pilot whale, respectively. However, infection patterns also suggest that a restricted level of colonization by parasites may also occur in other hosts via a shared diet. Due to the opportunistic nature of our research, we were unable to assess the relative roles of horizontal and vertical transmission of lungworms in the host species; however, we have provided new insights into the contributions and constraints of each in determining lungworm infection patterns in their cetacean hosts.

## Supplementary Information

**Additional file 1: Table S1.** Uncorrected pairwise distances between samples of the lungworm *Halocercus delphini* collected from different host species calculated with MEGA7. Dashes represent null values and repeat values.

**Additional file 2: Table S2.** Frequency of occurrence of prey taxa shared between 5 odontocete species from the western Mediterranean. ‘No. of prey taxa’ refers to the total number of prey taxa identified up to genus level, and ‘No. of shared prey taxa’, the number of taxa that are shared with any of the other species. Data obtained from Blanco et al. ([34, 35], unpub. data), Santos et al. [37] and Aznar et al. [36].

**Additional file 3: Table S3.** Published field surveys that provide quantitative data on the lungworm species detected in the present study.

**Additional file 4: Table S4.** Values of prevalence (%) for the three lungworm species found in this study in previous field surveys involving ≥ 3 host species. Hyphens indicate that the host species was analyzed but the lungworm species was not found. Numbers in parentheses indicate sample sizes. Abbreviations: Ref., Reference; Dd, *Delphinus delphis*; Fa, *Feresa attenuata*; Gg, *Grampus griseus*; Gm, *Globicephala melas*; Gma, *Globicephala macrorhynchus*; La, *Lagenorhynchus albirostris*; Lea, *Leucopleurus acutus*; Lh, *Lagenodelphis hosei*; Oo, *Orcinus orca*; Pe, *Peponocephala electra*; Sa, *Stenella attenuata*; Sb, *Steno bredanensis*; Sc, *Stenella coeruleoalba*; Sf, *Stenella frontalis*; Sl, *Stenella longirostris*; So, *Sagmatias obliquidens*; Sob, *Sagmatias obscurus*; Tt, *Tursiops truncatus*.

## Data Availability

Data used in analyses are available in the tables and supplementary files. ITS2 sequences obtained in this study have been uploaded to GenBank, and their accession numbers are available in Table 1.

## Abbreviations

ITS2: Second internal transcribed spacer
ML: Maximum likelihood
BI: Bayesian inference
PS: Pairwise percent similarity index
