# Supplementary material for: Determinants of lungworm specificity in five cetacean species in the western Mediterranean
Source: Parasit Vectors. 2021 Apr 12;14:196. doi: 10.1186/s13071-021-04629-1 (PMC8042974; doi:10.1186/s13071-021-04629-1)
Supplement: Supplementary file 1 — Additional file 1: Table S1. Uncorrected pairwise distances between samples of the lungworm Halocercus delphini collected from different host species calculated with MEGA7. Dashes represent null values and repeat values. [file 13071_2021_4629_MOESM1_ESM.docx]

**Additional file 1: Table S1.** Uncorrected pairwise distances between samples of the lungworm *Halocercus delphini* collected from different host species calculated with MEGA7. Dashes represent null values and repeat values.

|  | **Sequence** | **1** | **2** | **3** | **4** | **5** | **6** | **7** | **8** | **9** | **10** | **11** | **12** | **13** |
| --- | --- | --- | --- | --- | --- | --- | --- | --- | --- | --- | --- | --- | --- | --- |
| **1** | *Halocercus delphini* Gg | - | - | - | - | - | - | - | - | - | - | - | - | - |
| **2** | *Halocercus delphini* Tt | 0.048 | - | - | - | - | - | - | - | - | - | - | - | - |
| **3** | *Halocercus delphini* Dd1-1 | 0.060 | 0.012 | - | - | - | - | - | - | - | - | - | - | - |
| **4** | *Halocercus delphini* Dd2-1 | 0.060 | 0.012 | 0.024 | - | - | - | - | - | - | - | - | - | - |
| **5** | *Halocercus delphini* Sc1-1 | 0.024 | 0.024 | 0.036 | 0.036 | - | - | - | - | - | - | - | - | - |
| **6** | *Halocercus delphini* Sc2-1 | 0.048 | 0.000 | 0.012 | 0.012 | 0.024 | - | - | - | - | - | - | - | - |
| **7** | *Halocercus delphini* Sc3-1 | 0.048 | 0.000 | 0.012 | 0.012 | 0.024 | 0.000 | - | - | - | - | - | - | - |
| **8** | *Halocercus delphini* Sc3-2 | 0.048 | 0.000 | 0.012 | 0.012 | 0.024 | 0.000 | 0.000 | - | - | - | - | - | - |
| **9** | *Halocercus delphini* Sc3-3 | 0.048 | 0.000 | 0.012 | 0.012 | 0.024 | 0.000 | 0.000 | 0.000 | - | - | - | - | - |
| **10** | *Halocercus delphini* Sc3-4 | 0.036 | 0.012 | 0.024 | 0.024 | 0.036 | 0.012 | 0.012 | 0.012 | 0.012 | - | - | - | - |
| **11** | *Halocercus delphini* Sc4-1 | 0.048 | 0.000 | 0.012 | 0.012 | 0.024 | 0.000 | 0.000 | 0.000 | 0.000 | 0.012 | - | - | - |
| **12** | *Halocercus delphini* Sc4-2 | 0.048 | 0.000 | 0.012 | 0.012 | 0.024 | 0.000 | 0.000 | 0.000 | 0.000 | 0.012 | 0.000 | - | - |
| **13** | *Halocercus delphini* Sc4-3 | 0.048 | 0.000 | 0.012 | 0.012 | 0.024 | 0.000 | 0.000 | 0.000 | 0.000 | 0.012 | 0.000 | 0.000 | - |
| **14** | *Halocercus delphini* Sc4-4 | 0.048 | 0.000 | 0.012 | 0.012 | 0.024 | 0.000 | 0.000 | 0.000 | 0.000 | 0.012 | 0.000 | 0.000 | 0.000 |
