# Supplementary material for: Determinants of lungworm specificity in five cetacean species in the western Mediterranean
Source: Parasit Vectors. 2021 Apr 12;14:196. doi: 10.1186/s13071-021-04629-1 (PMC8042974; doi:10.1186/s13071-021-04629-1)
Supplement: Supplementary file 2 — Additional file 2: Table S2. Frequency of occurrence of prey taxa shared between 5 odontocete species from the western Mediterranean. ‘No. of prey taxa’ refers to the total number of prey taxa identified up to genus level, and ‘No. of shared prey taxa’, the number of taxa that are shared with any of the other species. Data obtained from Blanco et al. ([34, 35], unpub. data), Santos et al. [37] and Aznar et al. [36]. [file 13071_2021_4629_MOESM2_ESM.docx]

**Additional file 2: Table S2**. Frequency of occurrence of prey taxa shared between 5 odontocete species from the western Mediterranean. ‘No. of prey taxa’ refers to the total number of prey taxa identified up to genus level, and ‘No. of shared prey taxa’, the number of taxa that are shared with any of the other species. Data obtained from Blanco et al. ([34, 35], unpub. data), Santos et al. [36] and Aznar et al. [37].

|  | ***Globicephala melas*** | ***Grampus griseus*** | ***Tursiops truncatus*** | ***Delphinus delphis*** | ***Stenella coeruleoalba*** |
| --- | --- | --- | --- | --- | --- |
| **No. Dolphins** | 38 | 15 | 15 | 6 | 140 |
| **Total no. prey taxa** | 22 | 25 | 17 | 14 | 62 |
| **No. shared prey taxa** | 13 | 19 | 13 | 13 | 31 |
|  |  |  |  |  |  |
| **Cephalopods** |  |  |  |  |  |
| *Illex/Todaropsis* | 63.1 | 28.6 | 6.7 | 33.3 | 35.2 |
| *Sepia* | 28.9 | 21.4 | 6.7 | 16.7 | 1.5 |
| *Octopus* | 39.5 | 14.3 | 26.7 | 16.7 |  |
| *Sepiola* | 2.6 | 7.1 |  | 33.3 | 17.5 |
| *Todarodes* | 26.3 | 50 | 20.0 |  | 23.9 |
| *Eledone* | 76.3 | 28.6 | 13.3 |  | 2.3 |
| *Loligo* |  | 7.1 | 13.3 | 16.7 | 6.6 |
| *Histioteuthis* | 10.6 | 50 |  |  | 6.6 |
| *Brachioteuthis* |  | 28.6 |  |  | 22.5 |
| *Chiroteuthis* | 15.8 | 21.4 |  |  | 8.0 |
| *Mastigoteuthis* | 2.6 | 35.7 |  |  |  |
| *Ommastrephes* |  | 14.3 |  |  | 1.5 |
| *Ancistroteuthis* |  | 50 |  |  | 22.5 |
| *Onychoteuthis* |  | 28.6 |  |  | 16.6 |
| *Galiteuthis* |  | 7.1 |  |  | 2.9 |
| *Abraliopsis* |  | 7.1 |  |  | 23.1 |
| *Ancistrocheirus* |  | 21.4 |  |  | 9.3 |
| *Heteroteuthis* |  | 14.3 |  |  | 14.4 |
| *Ocythoe* |  | 21.4 |  |  | 0.7 |
| *Teuthowenia* | 18.4 |  |  |  | 0.7 |
| *Alloteuthis* |  |  |  | 16.7 | 11.5 |
|  |  |  |  |  |  |
| **Fish** |  |  |  |  |  |
| *Merluccius* | 2.6 |  | 73.3 | 16.7 | 29.4 |
| *Trachurus* | 2.6 |  | 20.0 | 16.7 | 7.5 |
| *Sardina* |  |  | 13.3 | 16.7 | 6.0 |
| *Micromesistius* | 2.6 |  |  |  | 8.1 |
| *Conger* |  |  | 40.0 | 16.7 |  |
| *Cepola* |  |  | 46.7 |  | 5.8 |
| *Ophidion* |  |  | 40.0 |  | 3.6 |
| *Phycis* |  |  | 20.0 |  | 3.6 |
| *Sardinella* |  |  |  | 16.7 | 3.6 |
| *Engraulis* |  |  |  | 16.7 | 4.5 |
| *Boops* |  |  |  | 33.3 | 14.0 |
