# Supplementary material for: Determinants of lungworm specificity in five cetacean species in the western Mediterranean
Source: Parasit Vectors. 2021 Apr 12;14:196. doi: 10.1186/s13071-021-04629-1 (PMC8042974; doi:10.1186/s13071-021-04629-1)
Supplement: Supplementary file 4 — Additional file 4: Table S4. Values of prevalence (%) for the three lungworm species found in this study in previous field surveys involving ≥ 3 host species. Hyphens indicate that the host species was analyzed but the lungworm species was not found. Numbers in parentheses indicate sample sizes. Abbreviations: Ref., Reference; Dd, Delphinus delphis; Fa, Feresa attenuata; Gg, Grampus griseus; Gm, Globicephala melas; Gma, Globicephala macrorhynchus; La, Lagenorhynchus albirostris; Lea, Leucopleurus acutus; Lh, Lagenodelphis hosei; Oo, Orcinus orca; Pe, Peponocephala electra; Sa, Stenella attenuata; Sb, Steno bredanensis; Sc, Stenella coeruleoalba; Sf, Stenella frontalis; Sl, Stenella longirostris; So, Sagmatias obliquidens; Sob, Sagmatias obscurus; Tt, Tursiops truncatus. [file 13071_2021_4629_MOESM4_ESM.docx]

**Additional file 4: Table S4**. Values of prevalence (%) for the three lungworm species found in this study in previous field surveys involving ≥ 3 host species. Hyphens indicate that the host species was analyzed but the lungworm species was not found. Numbers in parentheses indicate sample sizes. Host species abbreviations: Ref., Reference; Dd, *Delphinus delphis*; Fa, *Feressa attenuata*; Gg, *Grampus griseus*; Gm, *Globicephala melas*; Gma, *Globicephala macrorhynchus*; La, *Lagenorhynchus albirostris*; Lea, *Leucopleurus acutus*; Lh, *Lagenodelphis hosei*; Oo, *Orcinus orca*; Pe, *Peponocephala electra*; Sa, *Stenella attenuata*; Sb, *Steno bredanensis*; Sc, *Stenella coeruleoalba*; Sf, *Stenella frontalis*; Sl, *Stenella longirostris*; So, *Sagmatias obliquidens*; Sob, *Sagmatias obscurus*; Tt, *Tursiops truncatus*.

| **Ref.** | **Parasite** | **Host species** | | | | | | | | | | | | | | | | | |
| --- | --- | --- | --- | --- | --- | --- | --- | --- | --- | --- | --- | --- | --- | --- | --- | --- | --- | --- | --- |
|  |  | ***Dd*** | ***Sc*** | ***Sf*** | ***Sa*** | ***Sl*** | ***Lh*** | ***Tt*** | ***Gg*** | ***Gm*** | ***Gma*** | ***Fa*** | ***Pe*** | ***Sb*** | ***So*** | ***Sob*** | ***La*** | ***Oo*** | ***Lea*** |
| [38] | *Halocercus delphini* | 18.0  (50) | -  (8) |  |  |  |  | 10.0  (10) | -  (2) | -  (4) |  |  |  |  |  |  |  |  |  |
| [10] |  | 43.5  (101) | -  (14) |  |  |  |  | -  (3) |  | -  (3) |  |  |  |  |  |  | -  (6) | -  (1) | -  (5) |
| [45] |  | -  (7) | 33.3  (3) |  |  |  |  |  | -  (1) | -  (1) |  |  |  |  |  | -  (2) |  |  |  |
| [39] |  | 100  (3) | -  (1) |  |  |  |  | -  (2) |  | -  (3) |  |  |  |  |  |  | -  (3) |  | -  (3) |
| [42] |  | -  (66) | -  (31) |  | 5 (80) | 22.2  (18) |  |  |  |  |  |  |  |  | -  (13) |  |  |  |  |
|  |  |  |  |  |  |  |  |  |  |  |  |  |  |  |  |  |  |  |  |
| [38] | *Stenurus globicephalae* | -  (50) | -  (8) |  |  |  |  | -  (10) | -  (2) | 50.0  (4) |  |  |  |  |  |  |  |  |  |
| [10] |  | -  (101) | -  (14) |  |  |  |  | -  (3) |  | 33.3  (3) |  |  |  |  |  |  | -  (6) | -  (1) | -  (5) |
| [45] |  | -  (7) | -  (3) |  |  |  |  |  | 100  (1) | 100  (1) |  |  |  |  |  | -  (2) |  |  |  |
| [39] |  | -  (3) | -  (1) |  |  |  |  | -  (2) |  | 33.3  (3) |  |  |  |  |  |  | -  (3) |  | 33.3  (3) |
| [51] |  | -  (2) |  | -  (8) |  |  | -  (2) | -  (4) | -  (1) |  | 25.0  (4) | 25.0  (4) | 100  (1) | -  (1) |  |  |  |  |  |
|  |  |  |  |  |  |  |  |  |  |  |  |  |  |  |  |  |  |  |  |
| [39] | *Stenurus ovatus* | -  (3) | -  (1) |  |  |  |  | 50.0  (2) |  | -  (3) |  |  |  |  |  |  | -  (3) |  | -  (3) |
